# Organization of DNA in Mammalian Mitochondria

**DOI:** 10.3390/ijms20112770

**Published:** 2019-06-05

**Authors:** Géraldine Farge, Maria Falkenberg

**Affiliations:** 1 Centre National de la Recherche Scientifique/Institut National de Physique Nucléaire et des Particules, Laboratoire de Physique de Clermont, Université Clermont Auvergne, 63178 Clermont-Ferrand, France; 2 Department of Medical Biochemistry and Cell Biology, University of Gothenburg, 40530 Gothenburg, Sweden; maria.falkenberg@medkem.gu.se

**Keywords:** mitochondrial DNA, mtDNA compaction, mtDNA maintenance, mtDNA replication, mtDNA transcription

## Abstract

As with all organisms that must organize and condense their DNA to fit within the limited volume of a cell or a nucleus, mammalian mitochondrial DNA (mtDNA) is packaged into nucleoprotein structures called nucleoids. In this study, we first introduce the general modes of DNA compaction, especially the role of the nucleoid-associated proteins (NAPs) that structure the bacterial chromosome. We then present the mitochondrial nucleoid and the main factors responsible for packaging of mtDNA: ARS- (autonomously replicating sequence-) binding factor 2 protein (Abf2p) in yeast and mitochondrial transcription factor A (TFAM) in mammals. We summarize the single-molecule manipulation experiments on mtDNA compaction and visualization of mitochondrial nucleoids that have led to our current knowledge on mtDNA compaction. Lastly, we discuss the possible regulatory role of DNA packaging by TFAM in DNA transactions such as mtDNA replication and transcription.

## 1. DNA Compaction

In all forms of life, DNA is organized into a highly condensed structure to fit within the limited volume of the nucleus or cell. In order to structure the genome in such compacted configurations, cells make use of DNA supercoiling, macromolecular crowding, and a range of architectural basic proteins. Despite a general lack of homology between architectural proteins among organisms, the mechanisms by which they condense the genome appears to be highly conserved and falls into three categories: bending, wrapping, and bridging of DNA [1,2]. In bacteria, these architectural proteins are referred to as nucleoid-associated proteins (NAPs) or bacterial chromatin proteins [3]. In this category are only proteins that are responsible for the dynamic spatial organization of the DNA. Replication and transcription factors are not considered to be NAPs, even though they do transiently associate with chromosomal DNA. In general, NAPs are highly conserved within specific bacterial families and all bacterial species encode at least one NAP [4]. The bacterial NAPs act together to organize and compact the bacterial chromosome into a structure referred to as the nucleoid. Apart from structuring DNA into nucleoids, these proteins also play an important role in homeostasis of DNA supercoiling and in global gene regulation and gene silencing [5].

**Figure 1:**
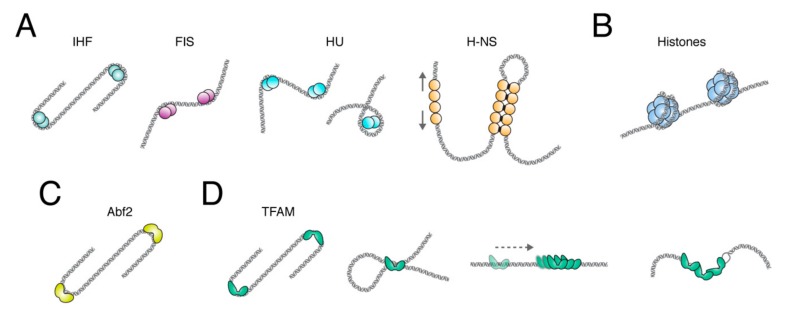
Architectural protein activities from bacterial nucleoids, eukaryotic chromatin, and mitochondrial nucleoids. (**A**) The bacterial chromosome is organized by several different NAPs that bend (IHF, FIS, HU), wrap (HU), and stiffen (arrows) and bridge (H-NS) DNA (grey duplex). (**B**) In the eukaryotic nucleus, chromosomal DNA is tightly wrapped around a nucleosome comprised of a histone octamer. (**C**) In yeast mitochondria, mtDNA is compacted by Abf2, which induces sharp U-turns. (**D**) In mammalian mitochondria, TFAM compacts mtDNA via U-turns and bridging (two left panels). In addition, TFAM slides along DNA (dashed arrow) and binds cooperatively to a TFAM patch, as well as increases DNA flexibility via local denaturation (DNA bubble, right panel).

The DNA-binding properties of NAPs are fundamental to their role in DNA compaction. Upon binding to DNA, they can bring distant domains into proximity or connect two distinct parts of the chromosome via bridge formation [6]. The most abundant and well-studied bacterial NAPs are DNA-bending and DNA-bridging proteins: histone-like protein (HU), histone-like nucleoid structuring protein (H-NS), factor for inversion stimulation (FIS), and the integration host factor (IHF) [7,8,9,10] (Figure 1A).

HU is among the most conserved NAPs in eubacteria, while H-NS, FIS, and IHF are found only in *Escherichia coli* and related enterobacteria [6]. HU is a small, very abundant NAP (18 kDa). There are two HU subunits, α and β, and, depending on the growth phase in *E. coli*, HU forms homodimers by self-association of HUα or heterodimers by interaction between HUα and HUβ [11]. HU inserts conserved proline residues into the minor groove of DNA, inducing a sharp bend in the DNA (Figure 1A). It has been suggested that HU can also wrap chromosomal DNA around itself [12] or, depending on the concentration, either induce flexible bends or form a rigid nucleoprotein filament [1]. H-NS is a another small (15.5 kDa) protein that shapes the bacterial nucleoid by bridging DNA, i.e., by bringing loci separated at the primary sequence level into close physical contact [2,13,14]. Bridging of different portions of DNA by H-NS has been directly demonstrated with both optical tweezers and atomic force microscopy experiments [2,14]. In addition, a divergent binding mode has also been suggested for H-NS, where the DNA adopts a stiffer configuration upon H-NS binding (the “stiffening mode”) [15,16] (Figure 1A). H-NS forms dimers via its N-terminal domain and can, at high concentrations, self-associate to form higher-order oligomers [17]. This oligomerization of H-NS enables the silencing of genes that are involved in virulence functions and genes that have been acquired by horizontal transfer, which are often more AT-rich than host chromosomal DNA [18,19]. However, a selective de-repression can occur when transcription from a neighboring region invades an H-NS bound locus, which disrupts H-NS repression [20,21].

Super-resolution microscopy data and the use of chromosomal conformation capture (3C) have led to important advances in our knowledge of bacterial nucleoid organization and its relation to transcription regulation [22]. Using fluorescence microscopy and fusion proteins, it has been shown that, in vivo, four of the five main NAPs are scattered throughout the nucleoid (HU, FIS, IHF, and StpA) [10]. The scattered localization of the NAPs supports the idea of there being a generic role for these proteins in DNA organization. Concerning 3C data, these have led to the elaboration of a model of the nucleoid arrangement of *C. crescentus* and *B. subtilis* with a helicoidal structure and an organization into subdomains [23,24]. However, these data were obtained from *C. crescentus* and *B. subtilis* and, to our knowledge, no 3C data are yet available for *E. coli*. It has been known for a long time that the structure of nucleoid and global transcription patterns are modified according to environmental conditions [25]. The structure of *E. coli* nucleoids undergoes changes during cell growth. In the stationary phase, the nucleoid is more tightly compacted than in the exponential phase [26]. These changes in nucleoid compaction and gene expression have, in part, been attributed to changes in the expression levels of NAPs [3,12]. For instance, there is a variation in the concentration of NAPs during cell growth, with the proteins FIS, HU, and H-NS being more abundant during the growth phase of the bacteria [6,26].

In the eukaryotic nucleus and in most archaea, packaging and compaction of the DNA is mostly achieved by histones (Figure 1B). DNA is wrapped around histones to form a nucleosomal fiber. This structure folds into a chromatin fiber, which is further organized into chromatin loops by proteins such as cohesin and the CCCTC-binding factor (CTCF) [27]. Apart from the nuclear DNA, a mammalian cell contains multiple copies of mitochondrial DNA (mtDNA), which is a circular molecule of ~16.5 kb in humans that only encodes 13 subunits of the respiratory chain, 2rRNAs, and 22tRNAs [28]. Assuming that each base pair occupies 0.34 nm in length, human mtDNA has a length of approximately 5.6 µm and, therefore, must be compacted in order to fit within a mitochondrion, which typically has a size one order of magnitude smaller. As with chromosomal and bacterial DNA, mtDNA molecules are not present (as initially believed) in “naked” or unprotected form, but are organized into small protein-DNA complexes called nucleoids.

## 2. The Mitochondrial Nucleoid

Mitochondrial nucleoids in human cells were first observed in the 1990s by epifluorescence after staining of DNA with the fluorochrome DAPI [29]. Subsequent studies on other cell types or tissues have confirmed that mtDNA is localized in discrete punctae―or mtDNA foci―throughout the mitochondrial network. These appear to be associated with the inner mitochondrial membrane [30]. These foci were called mitochondrial nucleoids, a name coined by analogy with the organization of the bacterial chromosome. The first nucleoid purification method identified two mtDNA binding proteins, which are the mitochondrial single-stranded DNA-binding protein (mtSSB) and transcription factor A (TFAM) [31,32]. Following these first studies, numerous attempts have been made to identify nucleoid-associated proteins by mass spectrometry (MS), using either purified native or cross-linked nucleoids [33,34,35] or immunoprecipitation of known nucleoid proteins [35,36,37,38]. The key components of the mitochondrial replication machinery (POL γ, Twinkle, and mtSSB) and the mitochondrial transcription machinery (POLRMT, TFAM, TFB2M, and TEFM) were among the most frequently identified proteins. However, other proteins such as mitochondrial ribosomal proteins, proteases, chaperones, RNA-binding proteins, and RNA processing proteins have also been found associated with the nucleoid. These approaches have also retrieved several contaminants, including even cytosolic and nuclear proteins [33,35], and have probably missed transient interaction partners that do not survive cell lysis and detergent washes. As a consequence of these technical issues, there is a lack of consensus regarding the exact molecular composition of the mitochondrial nucleoid-associated proteins. Recently, the mitochondrial nucleoid has also been studied using proximity-dependent labeling methods (biotin ligase-based (BioID) or peroxidase-based (APEX)) to tag endogenous nucleoid-associated components and identify their interacting partners in live cells. By using a Twinkle-APEX2 construct, 37 nucleoid proteins were identified by proteomic analysis. Of these proteins, 30 were known nucleoid proteins and 12 had functions related to RNA [39]. Furthermore, this technique appears to retrieve less cytosolic and nuclear contaminants than immunoprecipitation/fractionation techniques. However, this could be due to the more selective filters used here, as some proteins retrieved with immunoprecipitation/fractionation, such as LONP1 or MGME1, were not found in the APEX study. This technique, therefore, appears to be complementary to the immunoprecipitation/fractionation approaches and each can retrieve a unique set of nucleoid proteins.

For a long time, the detailed study of the nucleoid structure has been challenging due to the small size of the nucleoid and the inability of conventional fluorescence microscopy to resolve structures smaller than ~200 nm. The recent development of super-resolution techniques has enabled a better characterization of nucleoid size, shape, and organization, and also a more precise counting of mtDNA molecules present within a nucleoid. Several high-resolution microscopy techniques such as FPALM [fluorescence photoactivation localization microscopy], dSTORM [direct stochastic optical reconstruction microscopy], SIM [structured illumination microscopy], and STED [stimulated emission depletion] microscopy, and also cryo-electron tomography and rotary-shadowing electron microscopy have been employed [40,41,42,43,44,45]. These techniques have allowed the demonstration that nucleoids are rather small structures, with a mean size of ~110 nm [40,41,42]. Moreover, a consensus has emerged regarding the shape of the mammalian mitochondrial nucleoid, which appears to be slightly ellipsoid, with an average shape varying between the studies from slightly elongated (80 × 80 × 100 nm) [43] to truly ellipsoid (25 × 45 × 100 nm) [42,44]. Lastly, concerning the number of mtDNA molecules per nucleoid, nucleoids reconstituted in vitro and also nucleoids from mouse embryonic fibroblasts typically contain a single copy of mtDNA [43], even though there have been some findings supporting the idea of multiple mtDNA copies per nucleoid [45].

## 3. mtDNA Compaction in Yeast

**Figure 2:**
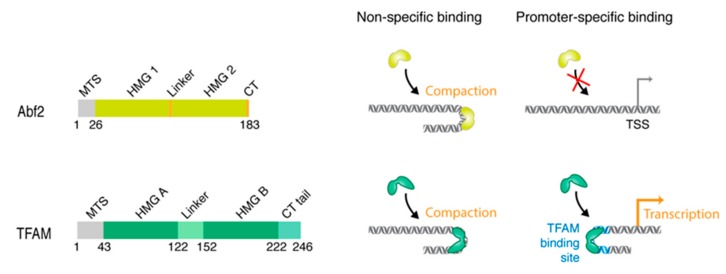
Comparison of yeast Abf2 and human TFAM. Left panels: Protein architecture of Abf2 (yellow) and TFAM (green) showing the mitochondrial targeting sequence (MTS), HMG boxes A and B, linker, and C-terminus. Middle panels: Abf2 and TFAM bind to DNA (grey duplex) non-specifically and induce compaction. Right panels: Abf2 has no promoter-specific binding activity and does not function as a transcription factor. Mammalian mitochondrial promoters have TFAM binding sites (blue duplex) to which TFAM can bind specifically and activate transcription. TSS- transcription start site.

Important insights into mammalian mitochondrial nucleoids and mtDNA compaction have come from studies on budding yeast [46]. In *Saccharomyces cerevisiae*, there are ~50 copies of mtDNA per cell, which corresponds to ~15% of the total cellular DNA content. Yeast mtDNA is a 24 to 29 μm molecule, which is mainly linear [47], and is compacted into nucleoid structures by the ARS- (autonomously replicating sequence-) binding factor 2 protein (Abf2p) [48]. Abf2p belongs to the high-mobility group domain (HMG) family of proteins (Figure 2).

The name Abf2p was coined because it was first identified as interacting with ARS, which is a nuclear chromosomal origin of DNA replication in yeast. However, it was later shown that Abf2p is found in mitochondria where it plays an essential role in mtDNA maintenance, since deletion of its gene causes rapid loss of mtDNA [49]. Abf2p is a very abundant protein and has a footprint of ∼27 bp on mtDNA [48]. Optical trapping of single DNA molecules extended by flow and visualized by fluorescence microscopy―and also atomic force microscopy (AFM) of Abf2p-DNA complexes―have shown that Abf2p can compact DNA [50,51]. The optical trapping experiment performed on double-stranded linear DNA provided information about the kinetics of binding of Abf2p to DNA and the force with which the Abf2p-DNA complex is packaged [50]. It was suggested that the compaction of DNA by Abf2p was rather weak, with a fast off-rate and small force stabilizing the DNA-protein complex. These AFM experiments have shown that the binding of Abf2p to both linear and circular DNA induces strong bends in the DNA backbone. The degree of DNA compaction was estimated by measuring the end-to-end distance of the DNA using increasing concentrations of Abf2p. At high concentrations of Abf2p, DNA was folded into a tight nucleoprotein complex. The authors derived a model from their experiments that suggested the sharp bends formed in the DNA backbone would be sufficient to cause DNA compaction. Recently, the group of M. Sola solved the crystallographic structure of Abf2p bound to an AT-rich double-stranded DNA [52]. They found that each HMG box of Abf2p induces a 90° bend in the DNA, which causes an overall U-turn. Moreover, DNA binding by Abf2p is modulated by the position of poly-adenine tracts: A-tracts have a narrow minor groove that make them inaccessible for Abf2p binding. This selective binding mediated by the DNA structure was proposed to be similar to the mechanism of nucleosome positioning in nuclear chromatin [53]. Overall, the picture that emerges is that mtDNA compaction in yeast is achieved only by the protein Abf2p through a rather simple mechanism that involves sharp bending of the DNA backbone and U-turn formation (Figure 1C). Whether or not other proteins are involved in mtDNA compaction in vivo still remains to be elucidated.

## 4. mtDNA Compaction in Mammals

As mentioned previously, the molecular composition of the mammalian mitochondrial nucleoid is still under debate and it has been suggested that most of the proteins involved in the different processes, such as transcription and replication factors, are only temporarily associated with the nucleoid. The only protein commonly recognized as a core component of the mammalian nucleoid is the mitochondrial transcription factor A (TFAM) [54]. TFAM is a very abundant protein, present in about 1000 molecules per mtDNA molecule or 1 TFAM protein molecule per 16–17 bp of mtDNA in mammalian cells. In other words, TFAM is abundant enough to coat the entire mitochondrial genome [41] and has the capacity to introduce negative supercoils in DNA [55]. TFAM is a 24-kDa basic protein with two HMG-box domains (HMGA and HMGB) that both intercalate into the minor groove of the DNA duplex [56,57]. HMGA and HMGB are separated by a linker, and HMGB is linked to a C-terminal tail (Figure 2). TFAM was first identified as a transcription factor that binds specifically to the promoter region of mtDNA and, together with the transcription factor B2, enhances transcription by the mitochondrial RNA polymerase (POLRMT) [58]. The structure of the initiation complex showed that the HMGB domain of TFAM is responsible for the interaction with POLRMT, which anchors POLRMT to the promoter [59]. In contrast, Abf2p does not have any role in transcription but is solely dedicated to organization and packaging of mtDNA. The C-terminal tail of TFAM appears to be essential for activation of transcription and is not present in Abf2p, which could explain why Abf2p has no transcriptional role in yeast (Figure 2). Initiation of transcription might not be the only role of TFAM in mtDNA metabolism, since it has also been suggested that TFAM might have a role in DNA repair [60] and it can bind to RNA-containing four-way junctions [61] and to specific DNA structures―G quadruplexes. The role of these nucleic acid binding activities remains to be determined [62]. Lastly, as with its yeast orthologue Abf2p, TFAM has an essential role in the organization of the mitochondrial genome. For this role, as opposed to its role in transcription initiation where it interacts specifically with the promotor regions, TFAM shows strong non-sequence-specific DNA binding. This specific and unspecific binding to DNA is an interesting feature of TFAM. Positioning of TFAM at specific sequence elements could also be important at sequences outside the promoter regions, which could lead to ordered binding of TFAM and DNA compaction throughout the entire mitochondrial genome. Such a regulated packaging could play a role in the control of mitochondrial gene expression and mtDNA replication.

To investigate the compaction by TFAM, different techniques of visualization and/or DNA manipulation have been used. However, the mechanisms by which TFAM mediates DNA compaction are still not completely understood. Similarly to Abf2p, one mechanism proposed for DNA compaction by TFAM is through bending of the DNA backbone (Figure 1D). The structure of TFAM bound to the promoter region of mtDNA has been solved. It has revealed that each of the two HMG-box domains of TFAM causes the DNA to bend almost 90°. This results in a complete 180° U-turn, which is a favorable arrangement for transcription initiation since it allows the TFAM C-terminal tail, which recruits the transcription machinery, to approach the initiation site, despite contacting a distant DNA sequence. Subsequently, it has been shown that TFAM also imposes a U-turn on DNA when bound unspecifically [63]. Recently, single-molecule FRET (fluorescence resonance energy transfer) experiments on TFAM/LSP complexes have shown that the DNA U-turn is induced by progressive and cooperative binding of the two TFAM HMG-box domains and the linker between them [64]. Other FRET data also argue in favor of a bending mechanism being responsible for compaction of unspecific DNA by TFAM [56,63,65]. However, other mechanisms have also been suggested. For instance, using a combination of optical tweezers and fluorescence microscopy, it has been shown that human TFAM can bind cooperatively to DNA sequences non-specifically and forms stable protein patches (filaments) in which each monomer covers about 30 bp of DNA. The binding of TFAM increased the flexibility of the DNA, likely via local base-pair melting of DNA (flexible-hinge mechanism), and provided an effective means of compacting mtDNA [66,67]. Lastly, data from electron microscopy and AFM have suggested that looping and cross-linking of DNA also play a role in mtDNA compaction [43,68]. Kukat et al. used low-angle rotary-shadowing EM to show a progressive compaction of DNA at increasing TFAM concentrations. During this compaction process, they observed cooperative binding of TFAM and also single TFAM molecules that were bridging neighboring DNA duplexes. Similarly, the AFM data of Kaufman et al. suggested that the compaction of DNA by TFAM involves bending of the DNA backbone and DNA loop formation, until the DNA is fully compacted. TFAM has been shown to interact with itself and form dimers in vitro and in vivo [63,69,70,71], seemingly via HMGA. The role of the dimerization of TFAM in DNA compaction has been examined. Using tethered particle motion (TPM) and a dimer mutant with five substitutions (K95A, Y99F, E106A, E112A, and R116A), Ngo et al. have come to the conclusion that dimerization is necessary for compaction but not for initiation of transcription [63]. However, electron microscopic images showed that the same mutant was able to compact DNA to the same extent as the wild-type protein [43]. Concerning the basic C-terminal tail of TFAM, it does not refer to a loose tail but includes highly structured regions that interact with the transcription machinery [59] and it is well established that this domain is essential for the promoter-specific transcription initiation. However, it has also been reported that truncation of the C-terminal tail decreased DNA-binding activity of TFAM by three orders of magnitude, which suggests that the C-terminal tail of TFAM is important for the strong general binding to mtDNA and could influence DNA compaction [72]. TPM experiments also showed that a truncated version of TFAM lacking the C-terminal tail retained the ability to compact DNA, but, to a lesser extent than wild-type TFAM [66]. The precise involvement of the C-terminal domain of TFAM in DNA compaction is, thus, a point that needs further clarification.

## 5. The Regulatory Role of TFAM

### 5.1. mtDNA Replication and Transcription

Before assessing the regulatory role of TFAM in mtDNA transactions, we will consider mtDNA replication and transcription. Mammalian mtDNA is replicated by proteins distinct from the nuclear DNA replication machinery, and several are related to replication factors identified in bacteriophages [73]. In human cells, DNA polymerase γ (POL γ) is the replicative DNA polymerase. POL γ is a heterotrimer consisting of one catalytic subunit (POL γA) and two accessory subunits (POL γB). At least four additional polymerases (PrimPol, DNA polymerase β, DNA polymerase θ, and DNA polymerase ζ) have been suggested to play a role in mitochondria [74]. However, none of them can substitute for POL γ [74]. At the mitochondrial replication fork, POL γ works together with the mitochondrial DNA helicase Twinkle, with Twinkle traveling ahead of POL γ and unwinding the double-stranded DNA template. Twinkle forms hexamers and heptamers [75,76,77] and requires a fork structure to load and initiate unwinding. A fourth protein is required at the mitochondrial DNA fork, which is the mitochondrial single-stranded DNA-binding protein mtSSB. mtSSB binds to the newly synthesized single-stranded DNA, protects it from nucleases, and prevents secondary structure formation [78]. Moreover, mtSSB enhances DNA synthesis by stimulating the helicase activity of Twinkle and increasing the processivity of POL γ [73]. Mammalian mitochondria also contain specialized DNA transcription machinery. The mitochondrial RNA polymerase (POLRMT), together with TFAM and the mitochondrial transcription factor B2 (TFB2M), initiates transcription from the mitochondrial heavy-strand and light-strand promoters (HSPs and LSPs) in vitro [54]. Transcription elongation by POLRMT is stimulated by the mitochondrial transcription elongation factor (TEFM), which stabilizes the interactions between elongating POLRMT and template DNA [54,59].

Several models for mtDNA replication have been suggested [54]. According to the strand displacement model, mtDNA replication is initiated from two separate origins of replication, which includes the H-strand and L-strand origins of replication (OriH and OriL). First, DNA synthesis is initiated at OriH and continues in one direction to produce the nascent H-strand. After the replication machinery has synthesized about two-thirds of the H-strand, OriL becomes single-stranded and activated. OriL folds into a stem-loop structure and POLRMT initiates primer synthesis from a poly-dT stretch in the loop region. Primers produced in this manner are used by POL γ to start L-strand synthesis with the parental H-strand as a template. The H-strand and L-strand continue to be synthesized until two complete daughter molecules have been formed. At the end of replication, the daughter molecules are separated in a topoisomerase 3α-dependent process [79]. The mechanisms of initiation of replication from OriH have still not been completely worked out. It is known that the RNA primers required for initiation at OriH are produced by POLRMT [54]. Transcription initiated from LSP can produce full-length transcripts stretching from LSP to the TERF1 binding site, but the majority of LSP transcription events are prematurely terminated after ~120 nucleotides, which form stable R-loops. These nascent R-loops are subsequently processed by RNase H1 to generate 3′-ends that can be used by POL γ to initiate DNA synthesis [80]. The relative levels of R-loop formation and full-length transcription are, in turn, regulated by TEFM [81,82,83]. Important aspects of the OriH initiation process that still need to be addressed include the mechanisms of primer removal and replisome assembly. How different levels of TFAM compaction affect these processes remains unclear, but it is tempting to speculate that TFAM may function as an epigenetic regulator for some of these key events (see next section).

### 5.2. The Role of TFAM in the Regulation of mtDNA Replication and Transcription

**Figure 3:**
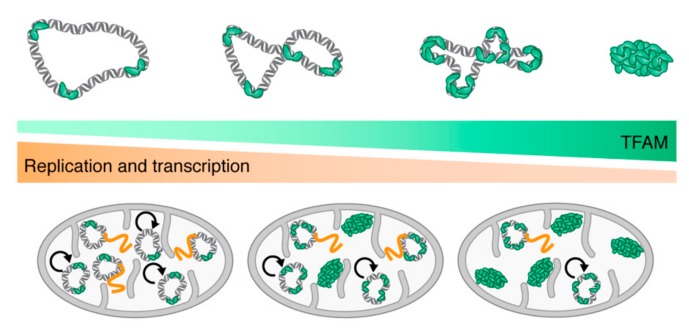
Regulatory role of TFAM in DNA transactions. Model of how TFAM levels and DNA compaction (upper panel) regulate replication and transcription in mitochondria (lower panel). Increases in TFAM levels (green) result in more DNA (grey duplex ring) compaction, which ultimately results in a fully compacted nucleoid. Mitochondria with low TFAM levels and, thus, less compacted mtDNA are permissive environments for replication (black arrow) and transcription (orange line). Increases in mitochondrial TFAM levels lead to more fully-compacted nucleoids that are refractory to replication and transcription, and fewer actively replicating/transcribing mtDNA molecules.

In all forms of life, DNA compaction is a means of regulating DNA transactions. As mentioned previously, H-NS is an important global regulator that regulates several hundreds of genes and that could act as an environmental sensor enabling physiological changes required for adaptation to different environmental conditions [84]. Similarly, in the nucleus, DNA compaction by histones into nucleosomes strongly influences gene transcription and DNA replication [85]. Nucleosomes are often precisely positioned at promoters and origins of DNA replication. Specific proteins are needed to change this positioning, which leads to gene activation and DNA replication. These proteins may act by modifying histones (e.g., acetylation or methylation) or by actively changing the position of individual nucleosomes. The mitochondrial nucleoids are much more structured entities than previously believed, and they may be regulated in a manner similarly to nuclear chromatin. In yeast, there is less Abf2 protein bound per molecule of mtDNA in transcriptionally active nucleoids [86]. In contrast, over-expression of Abf2p causes a rapid loss of mtDNA [87], which is a phenomenon that has been attributed to excessive DNA compaction. This would prevent the DNA replication machinery from gaining access to the DNA. The effects of increasing TFAM concentration and, thus, the effects of nucleoid formation on mtDNA replication and transcription have been monitored by using reconstituted nucleoid-like particles in vitro. It was found that small changes in TFAM levels dramatically affect the proportion of DNA molecules that are available for transcription and DNA replication (Figure 3).

In compacted nucleoids, TFAM forms stable protein filaments on DNA that block melting and prevent progression of the replication machinery and the transcription machinery. Based on these observations, it has been suggested that small variations in the TFAM-to-mtDNA ratio may be used to regulate mitochondrial gene transcription and DNA replication [88]. In vivo experiments have shown variable levels of colocalization between TFAM and foci of BrdU incorporation. This has also led to the suggestion that the population of BrdU-positive, TFAM-negative foci could represent actively replicating templates, which could subsequently acquire additional TFAM and become compacted when replication is terminated [89]. Moreover, in vivo, super-resolution microscopy data have revealed different forms of mammalian nucleoids [43], which appears to corroborate the hypothesis that the more compact nucleoids are of an mtDNA storage form, whereas the larger forms are involved in active replication and/or transcription. TFAM may, therefore, function as an epigenetic regulator that controls the number of mtDNA molecules available for active transcription and/or mtDNA replication. Lastly, different post-translation mechanisms for regulation of binding of TFAM to DNA have been suggested. TFAM might be modified by acetylation [90] or phosphorylation [91] within the HMGA domain. The DNA binding and compaction capacities and also the speed of sliding on DNA of TFAM mutants that mimic the effects of acetylation or phosphorylation were examined. The differences obtained for these parameters between some of the mutants and the wild-type TFAM suggested that these modifications might be involved in the regulation of mtDNA transcription in vivo [92].

**Figure 4:**
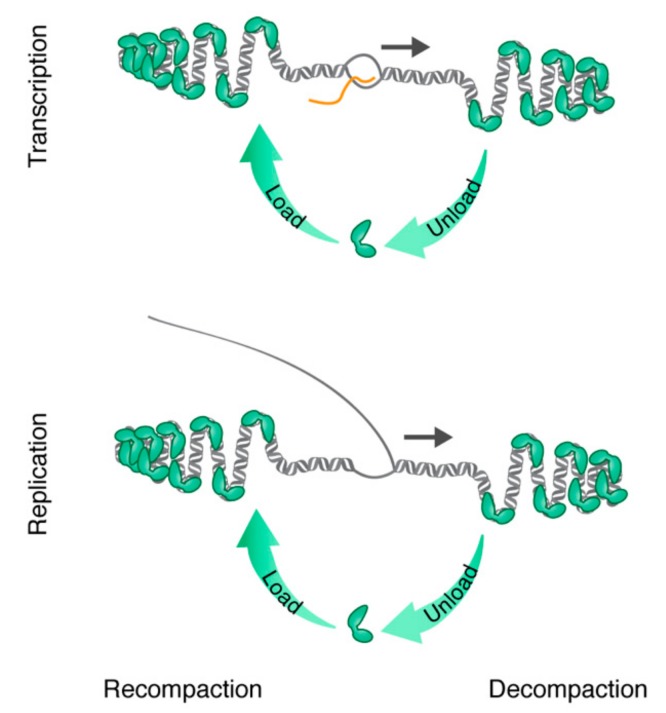
Dynamics of TFAM binding during transcription and replication. Model of how nucleoid decompaction and re-compaction facilitate transcription (upper panel) and replication (lower panel). TFAM (green) unloads from the DNA (grey duplex) ahead of the transcription bubble or replication fork to enable progression (dark arrows) of the transcription and replication machineries. TFAM may then reload directly behind in order to recompact the DNA.

As mentioned above, packaging of nuclear DNA into nucleosomes makes it partially inaccessible to the transcription machinery. A large number of different chromatin remodeling factors are required to disrupt the nucleosomal structure in a regulated manner, including histone modifiers and chaperones. It is tempting to speculate that specific remodeling factors are also required in mitochondria, in order to re-model TFAM-dependent packaging of mtDNA during replication and transcription (Figure 4). Alternatively, the relative high on and off rate of TFAM [66] could circumvent the requirements of remodeling factors. These intriguing possibilities will need to be addressed in future investigations.
